# Iod. Pot. in Hydrocephalus

**Published:** 1848

**Authors:** 


### ARTICLE III.

### IOD. POT. IN HYDROCEPHALUS.

In a private letter to one of the Editors, Dr. C. Brackett, of Rochester, Ind., makes the following remarks upon the use of hyd. pot. in hydrocephalus:

I have met with two distinctly marked cases which, under ordinary methods of treatment, I am satisfied, would soon have proved fatal, yet they recovered perfectly under the use of iod. pot.

If in other hands it proves as useful as I have found it, then truly is it invaluable. So soon as copious diuresis (and in one case salivation) had taken place, the little sufferer began to recover. In one case which I treated, last summer, I did not see the child till after paralysis and insensibility had taken place. The iod. was administered; an amendment was soon observable, and recovery took place rapidly, with the exception of the palsy of the left arm and leg which continued for a long time. I at length persuaded the parents to bring the child to town, when I administered strychinne internally. An immediate change for the better was observed; the patient recovered perfectly.

In the same letter from which the above extract is made, Dr. Brackett gives us the following history of a case, showing the importance of vaginal examinations in diagnosis:

I was called to see Mrs. W——, who for many years had been "weakly;" she had suffered for some time from repeated floodings, heavy dragging pains in the back and loins, inability to do much work, particularly that which requires one to lift, or be on the feet.

I suspected some organic difficulty, made an examination per vaginam (which she was very unwilling to submit to, and would not, had I not refused to do anything for her till I was fully satisfied as to the difficulty), and found growing from the os uteri, from the left side, an insensible fleshy growth about three inches long, an inch and a half in diameter at its lower part; much smaller at its attachment at the os. I took two pieces of wire, made a ring in the end of each, passed a small cord of silk through the rings; passed both wires up together by side of polypus, rings up, then separating one from the other, brought it round again to its fellow, thus encircling the neck of the excresence with the ligature; I then drew down the ends as tight as possible, twisted the wires, and left her for a few hours. She felt no pain, but flooded more for a few minutes after the ligature was tightened.

In fifteen hours, I pulled at the ligature, previously tightening and twisting it, when they, with the polypus, came away, much to the surprise of the patient. She soon got up, said she felt "lighter" than she had for six years previously; recovered rapidly, and now enjoys good health.

Are not many cases of polypus uteri treated as simple menorrhagia?

